# Viruses and cells intertwined since the dawn of evolution

**DOI:** 10.1186/s12985-015-0400-7

**Published:** 2015-10-16

**Authors:** Julia Durzyńska, Anna Goździcka-Józefiak

**Affiliations:** Department of Molecular Virology, Institute of Experimental Biology, Faculty of Biology, A. Mickiewicz University, ul. Umultowska 89, 61-614 Poznań, Poland

**Keywords:** Virus, Giant virus (girus), Evolution, LUCA, Horizontal gene transfer, Tree of life, Lineage, Domain of life

## Abstract

Many attempts have been made to define nature of viruses and to uncover their origin. Our aim within this work was to show that there are different perceptions of viruses and many concepts to explain their emergence: the virus-first concept (also called co-evolution), the escape and the reduction theories. Moreover, a relatively new concept of polyphyletic virus origin called “three RNA cells, three DNA viruses” proposed by Forterre is described herein. In this paper, not only is each thesis supported by a body of evidence but also counter-argued in the light of various findings to give more insightful considerations to the readers. As the origin of viruses and that of living cells are most probably interdependent, we decided to reveal ideas concerning nature of cellular last universal common ancestor (LUCA). Furthermore, we discuss monophyletic ancestry of cellular domains and their relationships at the molecular level of membrane lipids and replication strategies of these three types of cells. In this review, we also present the emergence of DNA viruses requiring an evolutionary transition from RNA to DNA and recently discovered giant DNA viruses possibly involved in eukaryogenesis. In the course of evolution viruses emerged many times. They have always played a key role through horizontal gene transfer in evolutionary events and in formation of the tree of life or netlike routes of evolution providing a great deal of genetic diversity. In our opinion, future findings are crucial to better understand past relations between viruses and cells and the origin of both.

## Background

Nowadays, to give a concise definition of virus nature is troublesome. Researchers of different standpoints have proposed several interpretations. Viruses by their nature seem to be entities somewhere in between inert and living worlds [1]. For decades viruses were simply considered as pathogenic biochemical entities composed of two major elements: nucleic acid (RNA or DNA) constituting their genome and protein coat (capsid). Many viral particles (virions) are even more complex and contain lipid-protein envelope or an additional capsid, and specific viral enzymes required for replication [2, 3]. On the other hand, viruses can also be considered as living organisms since upon infection of cells they turn them into virocells [4–6]. Moreover, a concept of a greater virus world has recently been formulated covering bona fide capsid-encoding viruses and other capsidless replicons such as plasmids, transpozons and viroids. The major feature of this world is not presence of a capsid but genetic, informational parasitism [7]. These capsidless replicons were also named orphan replicons [8]. Emergence of capsid coding sequences and proteins was a big evolutionary step as appearance of these vehicles to transfer and protect nucleic acids was one of prerequisites for evolution. A few years ago, a new division for all living organisms into two distinct groups has been proposed: ribosome-encoding organisms (REOs) and capsid-encoding organisms (CEOs) [8]. Similarly to viruses, life itself is also difficult to define and throughout history of science from Aristotle to K. Ruiz-Mirazo definition of life has been modified many times and since life is a process and not a substance, it is challenging to confine “life” in a simple, yet exhaustive formula. A very detailed timeline with changing definitions of life or living beings is nicely depicted by Moreira and Lopez-Garcia [9]. It is important to know these different explanations for the sake of further discussion presented herein.

The range of viral genome sizes spans three orders of magnitude and simple size-based distinction between viruses and cells valid for over a century cannot be used any longer after the discovery of giant viruses, also known as giruses [10]. One of the smallest double-stranded DNA (dsDNA) viruses is hepatitis B virus (HBV) containing a 3.2 kb genome with only several genes. Even smaller “subviral” partner of HBV is human hepatitis delta virus (HDV), quite similar to viroids in many regards. It contains a 1.7 kb genome encoding one antigen and shows ribozyme activity [11, 12]. On the other hand, the largest dsDNA viruses, Pandoraviruses, have genomes of 2.5 Mb encompassing some of 2500 coding sequences [13]. According to the Baltimore classification developed by David Baltimore in the early ‘70s, there are seven types of all known viruses depending on the nucleic acid content and its replication mode: dsDNA, ssDNA, dsRNA, (+)ssRNA and (−)ssRNA, ssRNA-RT and dsDNA-RT viruses, each with a different replication strategy within infected cells [14]. Cellular forms of life use the canonical DNA-RNA-protein replication-expression pattern, whereas viruses are totally dependent on cells for multiplication and exploit all possible DNA and RNA interconversions [15]. Retroviruses and hepadnaviruses can reverse transcribe their RNA to DNA and this process is rare to occur in cells, although exceptions have been recently reported such as telomere synthesis and presence of reverse transcriptase-related cellular genes in eukaryotes [16–18]. Eukaryotic telomeres essential for the linear chromosome organization most probably derived from an ancient retroviral activity [19].

Many researchers postulate that viruses are of polyphyletic origin and different RNA and DNA viruses derived independently as opposed to monophyletic cellular domains coming from one ancient ancestor LUCA (last universal common ancestor), which is a logical consequence of the binary mechanism of cell division [6]. There is no physical ‘fossil record’ of viruses; virions persist for short time periods, and rapidly degrade leaving no direct trace of their existence. However, many viral genomes have always had the capacity to integrate into cellular genomes and the study of this genomic ‘fossil record’, called paleovirology, helps to understand the long-term evolutionary history of virus–host interactions [20]. Viruses have been major players in the evolution by imposing high selection pressure on their hosts and manipulating the whole environment [21]. According to recent hypotheses, viruses might have played a direct role in the origin of DNA and DNA replication mechanisms [22], cellular envelopes [23], of pathogenicity [24], alternative genetic codes [25] and formation of the three domains of life: Archaea, Bacteria and Eukarya [23], which were identified by 16/18S rRNA comparison [26]. A previously described link between reverse transcriptase activity and telomeres indicates a possible early retroviral colonization of large dsDNA viruses, which are putative ancestors of the eukaryotic nucleus [19], although a different concept on the origin of nucleus was also reported [27].

With the advent of this new knowledge it was a bit unthoughtfully proposed by Carl Woese that cellular evolution could not be solely explained by the classical Darwinian mode of thinking [28]. However, it has been recently pointed out that the core of Darwin concept of evolution relying on variation/selection processes is still sufficient to explain the history of life. Horizontal, also called lateral gene transfer (HGT or LGT) should be considered a special case of genetic variation along with mutations, recombination, and different kinds of ploidies and others [29–31]. All these processes enrich biodiversity and influence cellular evolution [32], and thus HGT is supplementary to vertical evolutionary mechanisms. If a proto-cell was simple and highly modular in organization, it implies that HGT could have played a greater role in evolution [33]. This modularity of ancient RNA cells is somehow reflected by structure of current viral genomes built of major functional blocks of genes (modules): 1) replicon - ORFs involved in replication, 2) structural genes encoding coat proteins and 3) elements manipulating metabolism of infected cells. Phage genomes could be considered as collections of functional modules that evolved independently in host genomes and were acquired over time by the phage [34]. However, nowadays an overall similarity of viral and cellular proteins having probably resulted from horizontal gene transfer is small [35].

If we imagine that 1 ml of seawater contains 1 million bacteria cells and ten or even a thousand times more viral sequences (up to 10^9^ virions/ml), it can be determined that 10^31^ bacteriophages infect 10^24^ bacteria cells per second [36, 37]. This abundance and replication rate of viruses have also been one of the sources of novel functions in cellular lineages via the insertion of genes of viral origin into cellular genomes. It has been recently suggested that viruses are real nature’s genomic laboratory and a virocentric perspective on the evolution of life was put forward [15, 38, 39]. However, the viral insertion should be distinguished from the real HGT consisting of DNA exchange between cells by transformation, conjugation and transduction; in the latter viruses play the role of vehicles for cellular gene exchange [29]. These different processes can be exemplified by a prophage providing the acquisition of more than 100 new genes in a single genome editing event [40] or an insertion sequence named IS607 and carried by *Phycodnaviridae* (members of nucleo-cytoplasmic large DNA viruses, NCLDVs) as well as by Amoeba and Algae. It suggests that these viruses could mediate horizontal transfers between different cellular genomes [41]. The majority of sequences in viromes represent a so-called “dark matter”, they have no detectable homologues in the current databases [42]. In case of phages, it means that their ability to transduce cellular genes does not translate into domination of these cellular “hitchhiker” genes in the phage genomic reservoir [43]. Although the number of sequenced genomes now included in the databases dramatically increased in the last years, the percentage of unknown sequences within bacteriophages has not really decreased [39].

### Hypotheses of virus origin

**Fig. 1:**
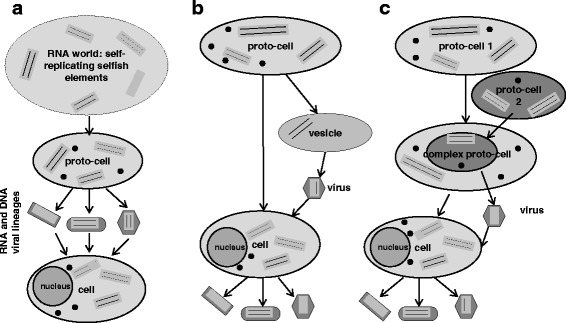
Three major theories of virus origin. Arrows show the direction of evolutionary changes. **a**. According to the virus-first hypothesis at the dawn of life there were no cellular forms but only first RNA molecules possessing enzymatic activities and capable of self-replication, also called selfish genetic elements. **b**. According to the escape hypothesis viruses derived from cellular RNA or/and DNA fragments such as plasmids and transpozons. During asymmetrical cell fission a vesicle (smaller cell-like entity) could have formed engulfing a self replicating RNA and a coat encoding RNA segment. **c**. According to the reduction hypothesis viruses come from small primordial cells (not necessarily primitive), which lost their cellular elements in the course of evolution. They maintained, however, their genetic material and certain elements needed for replication. Proto-cells presented in this picture already contained ribosomes (black small plain circles) and were able to produce proteins/capsids, whereas cells containing a nucleus correspond to modern cells, which descended from LUCA. Eukaryotic cells were used to depict all three hypotheses of virus origin and underline a possible involvement of viruses in eukaryogenesis

Viruses cannot multiply or carry out living processes outside the cells, therefore the ancestry of both is most probably highly intertwined. Origin of viruses is enigmatic and controversial in the light of cellular theory of life. Comparison of viral and cellular sequences shed more light on hypotheses of virus origin. It is important to know that evolutionarily, three classes of viral genes can be distinguished: 1) genes with detectable homologues in cellular life forms, 2) virus-specific genes such as ORFans and 3) viral hallmark genes with only distant homologues in cellular organisms [44]. None of the theories is exhaustive and each has gaps difficult to explain, and for each theory pros and cons have been discussed in literature (Fig. 1).

The virus-first (or co-evolution) hypothesis was first proposed by d’Herelle who claimed that viruses are ancestral to cells [45]. Others suggested that viruses originated in the pre-cellular world using a soup as a host [15, 46]. Evolution of life started with a virus-like stage and the advent of modern-type cells was a comparatively late event [15]. At the dawn of life there were no cellular forms but only first RNA molecules possessing enzymatic activities and capable of self-replication. Some of these subviral forms still exist in the current world - they are viroids – the smallest (from ~250 to ~400 nucleotides) and simplest replicating RNA molecules known today [47]. Viroids of the family *Avsunviroidae* possess a hammerhead ribozyme structure and can carry out cleavage of oligomeric forms of RNA to the monomeric forms, which potentially makes them descendants of the earliest biomolecules present on Earth [48, 49]. Viroids are good candidates for being survivors of the RNA world as they have a number of special features such as a small size imposed by error-prone replication, high G + C content, lack of protein-coding ability consistent with a ribosome-free habitat and several others. On the timeline of evolution, when DNA and proteins molecules already existed, those protoviroids (ancient viroids) lost some abilities to become the plant parasites and today they are dependent on cellular enzymes such as RNA polymerase, RNAaseH and RNA ligase for replication [49]. However, it is more reasonable to claim that these protoviroids would have always relied on efficient cellular metabolism producing ATP and other ribonucleotides, and therefore ancient viroids and cells co-evolved.

Even though we have no insight, whether there were the same rules in this ancient realm, according to our knowledge of the current living world there is a strong inverse relationship between genome size and mutation rate across all replication systems, therefore it is possible that pre-LUCA genomes were both small and highly error prone and hence RNA virus-like [50]. In the era of nucleic acid life in a niche of “supramolecular aggregates” (or SMAs), the nucleic acids evolved to accommodate available peptides [28], or RNA molecules could have evolved independently of host proto-cells as their “parasites”, inhabiting a common environment. RNA viruses evolved first from the nucleoprotein world, followed by retroid elements, and DNA viruses [44]. Ancient RNA viruses, specifically (+)ssRNA, are relics of the RNA world, retroviruses and hepatitis B viruses relics of an RNA-to-DNA transition in evolutionary history of life. This seems to be confirmed by the existence of tRNA-like structures (TLSs), which are involved in virus replication (link between replication and translation) and by the discovery of reverse transcriptase [51]. Transfer RNA-like structures (TLSs) that are sophisticated functional mimics of tRNAs are found at the 3′-termini of the genomes of a number of plant positive strand RNA viruses and three natural aminoacylation identities are represented: valine, histidine, and tyrosine [52]. Indeed, tRNA-like motifs could be inherited from RNA replication signals accommodated to assist in the translation process. Plant RNA viruses make use of TLSs to control translation initiation and viral RNA replication [53].

There is a growing body of evidence that viruses arose even before LUCA, that more appropriately should be denoted as Last Universal Cellular Ancestral State (LUCAS) [54]. Moreover, while discussing concepts of virus origin, it is crucial to distinguish viruses evolved before ancient cells and viruses evolved before modern cells, the descendants of LUCA. The theory of ancient virus origin is supported by the presence of homologous capsids and homologous packaging ATPases among diverse viruses infecting the three domains of life. Capsid protein is the most prominent example, and the sole protein found in most viruses and not in cellular organisms [51, 55, 56]. Several years ago Abrescia and colleagues identified major viral lineages based on structural comparison of non homologous capsid proteins and non homologhous packaging ATPases, where genomic similarities are no longer observable. At least two lineages of DNA viruses predating LUCA, adenovirus/PRD1 containing the double-jelly roll fold and the Hong Kong fold in the HK97 lineage were described [3]. In the context of these two completely different structures a criticism of the virus-first theory based on structural convergence of most viral capsids adopting to a small number of simple geometrical structures can be refuted. Thus, the convergence towards similar folds for adaptation of certain capsid proteins, as their tertiary conformation is subject to strong constraints, concerns only a part of the viral world and cannot be ground for a universal evolutionary concept. Furthermore, the invention of a self-assembling capsid is very difficult to achieve and its formation by evolutionary mechanisms is very rare. It suggests that structure based lineages may tend to reflect homology rather than structural convergence [3]. To conclude it should be noted that there is no a single gene, or a coding sequence that would be common to all the viruses, hence a common pre-LUCA viral ancestor is often questioned [9].

The escape or vagrancy (cell-first) hypothesis describes viruses as derived from cellular RNA or/and DNA fragments such as plasmids and transpozons, which escaped from cells. When such RNA or DNA fragments acquired protein coat they became independent entities capable of infecting cells from which they had escaped previously [57]. As already mentioned, ancient RNA genomes were modular (“RNA chromosomes”), and were randomly distributed from cells to cells [58]. During asymmetrical cell fission a vesicle (smaller cell-like entity) could have formed engulfing a self replicating RNA and a coat encoding RNA segment. The translation apparatus was not transferred to the newly formed vesicle and thus an ancient RNA virus emerged [51]. In a model for early virus evolution, viruses can be regarded less as having derived from proto-cells and more as being partners in their mutual co-evolution [59]. This model somehow merges the virus-first and the escape hypotheses into one more complex theory. On the other hand, it would be difficult to demonstrate how nucleic acids released from cells started to code for coat proteins. It can be easily imagined that plasmids evolved quite late from dsDNA viruses (not the other way round) and lost genes encoding coat proteins. Otherwise, it would be hard to prove how viruses evolved from plasmids and acquired the ability to encode capsids in the absence of already existing capsid modules [60, 61]. Furthermore, viruses resemble plasmids which do not encode cellular homologues including proteins involved in DNA replication such as rolling-circle Rep proteins and DNA polymerase E [62]. This would indicate common evolutionary tract for plasmids and viruses. Moreover, viruses derived from cells should share a high sequence homology with their hosts, yet proteins encoded by bacteriophage T4 are more similar to eukaryotic proteins or eukaryotic viral proteins than to their bacterial homologues [63], and most proteins encoded by viral genomes are deprived of their cellular homologues [64]. However, it is easier to defend the escape theory in the context of a pre-LUCA scenario for virus origin. Since viruses derived from genome fragments escaped from cells predating LUCA, any specific relationship between proteins encoded by viruses and those encoded by their hosts are not expected anymore [51]. A good documented example of new viruses being created through gene escape events is human hepatitis delta virus (HDV), which has been shown to contain a ribozyme sequence that is closely related to the CPEB3 ribozyme present in a human intron [65]. HDV is found only in humans and requires human hepatitis B virus to replicate. Thus, HDV probably derives from the human transcriptome, and not necessarily from a pre-LUCA world [50].

The reduction or degeneracy (cell-first) hypothesis states that viruses come from small primordial cells (not necessarily primitive), which lost their cellular elements in the course of evolution. They maintained, however, their genetic material and certain elements needed for replication. For a long time it has been believed that there is no intermediary form between a cell and a virus, because parasites known for the three domains of life have kept their cellular character; they still have ribosomes and are able to synthesize ATP. It is also for that reason that the reduction theory can be easily counter-argued. But then again, it is much easier to imagine this reduction leading to a virus emergence in a world of RNA cells, because these cells were much simpler than the modern ones. RNA-cell living as a parasitic endosymbiont in another RNA cell could have lost its own machinery for protein synthesis and for energy production, using instead those of the host [60]. The presence of virus hallmark genes may be considered as evidence for their possible origin from virocells or these sequences may have been recruited from ancient cells now extinct. For instance, it was described that both human adenovirus and *Bacillus subtilis* bacteriophage Ф29, use a similar atypical protein-priming mechanism to replicate their DNA (unknown in the cellular world) and encode a unique type of DNA polymerase from the subfamily of polymerases B. It can use such a DNA template to initiate its own replication and has no representatives in currently living cells [66]. It seems likely, that the DNA polymerase is a viral hallmark gene in disguise [44], and that these two viruses originated from a common ancestor that had existed before the divergence between Eukarya and Bacteria [51]. However, it must be mentioned here that a small set of virus hallmark genes encoding essential functions shared by a vast range of viruses is a strong evidence, especially for positive-strand RNA, that viruses are direct descendants of the primordial RNA-protein world [15].

In recent years, the reduction hypothesis was revived by the discovery and genomic characterization of *Acanthamoeba polyphaga* mimivirus (APMV) [67] with a very complex set of genes (1,2 Mb genome and 911 genes) showing little horizontal gene transfer. It strongly suggested a process of reductive evolution from an even more complex ancestor that had been endowed with a protein synthetic capability [57]. Furthermore, sequence and phylogenetic analyses of the components of the packaging machinery present in APMV show that some large DNA viruses such as mimivirus, vaccinia virus, and pandoravirus are remarkably more similar to prokaryotes (bacteria and archaea) than to other viruses in the way they process their newly synthesized genetic material to make sure that only one copy of the complete genome is generated and meticulously placed inside a newly synthesized viral particle [68]. The discovery of giruses such as Mimiviruses [10], Megaviruses (*Megavirus chilensis*) [69], Pandoraviruses [70], and Pithoviruses [71] created a continuum in genome size and functional complexity between the virosphere and cells. Megavirus retained all of the genomic features unique to Mimivirus, in particular its genes encoding key-elements of the translation apparatus (seven aminoacyl-tRNA synthetases), a trademark of cellular organisms. It could suggest that large DNA viruses derived from an ancestral cellular genome by reductive evolution, which can be supported further by the presence of a large number of enzymes in genomes of giruses like various hydrolases, proteases, kinases, phosphatases and many others involved in cellular metabolic processes. The nature of this cellular ancestor remains hotly debated [70–72]. It has been pointed out by Claverie and Ogata that despite life being an all or nothing concept, “living” organisms span a continuum of autonomy and complexity in which large DNA viruses (giruses) largely overlap the smallest bacteria. It is a well described evolutionary scenario for Bacteria and Archaea to become parasites by reductive evolution. Since giruses could have predated the divergence of today’s three cellular domains, their case may be similar supported by the presence of bacterial-like, archaeal-like and eukaryan-like genes in their genome [73]. That is why it has recently been proposed that giruses coexisted with the cellular ancestors and represent a distinct supergroup along with superkingdoms Archaea, Bacteria and Eukarya [74].

However, this evolutionary theory suffers from several major weaknesses. More than 93 % of Pandoraviruses genes resemble nothing known in all available sequence databases, therefore their origin cannot be traced back to any known cellular lineage [70], quite similarly to previously described bacteriophages. Furthermore, the term “fourth domain” is controversial and many arguments were given by opponents against viruses belonging to the tree of life (actually, the tree of cells), among others, inability to produce and capture energy or inexistence of integrated fully developed metabolic pathways [6, 9]. NCLDVs genomes do not display any characteristics of genome decay that have been observed in intracellular bacteria such as Rickettsia or parasitic protists such as microsporidia, where presence of pseudogenes, non-coding DNA, shorter genes, massive gene loss and disappearance of metabolic pathways were noted. This picture is blurred even more by the fact that Megaviruses are related to small DNA viruses and could have derived from them using a complex process of genomic accordion. It implies successive steps of genome expansions (duplication and gene transfers) and genome reduction, in addition to movement and amplification of diverse genetic elements [75]. Furthermore, giruses can be infected by their own viruses called virophages such as Sputnik that could be a vehicle mediating lateral gene transfer between them [76]. As Sputnik multiplies in giant factories, it resembles satellite viruses of animals (adeno-associated virus or hepatitis D virus). However, Sputnik reproduction cycle seems to impair the production of normal APMV virions significantly, indicating that it is a genuine parasite, a first virus described to propagate at the expense of its viral host [76]. According to Krupovic and Koonin Megaviruses evolved from virophages, which in turn derived from Polintons and *Tectiviridae* as it is shown by homology of the major capsid protein (MCP) in these groups. The evolution of giant viruses had been pushed to the extreme, which explains their big genome size [3, 77, 78]. To conclude, one should avoid supporting the reduction concept of virus origin using NCLDVs biology.

### The origin of cells and nuclei

The co-evolution of viral elements and cellular forms has also been described as incessant arms race with various forms of cooperation [79]. It started 3 or 4 billion years ago, when LUCA also emerged [80] to give life to all cellular organisms we know nowadays with universal genetic code from bacterial to human cells, wherein basic processes are similar. To reconstruct LUCA as it was back in time is extremely difficult because organisms have lost many genes in the course of evolution, and additionally a horizontal gene transfer (HGT) interfered. The very nature of LUCA is still under discussion. According to a group of researchers, although it does not seem very likely in the light of more robust theories, LUCA could have been an inorganically housed assemblage of expressed and replicable genetic elements. The evolution of the enzymatic systems for DNA replication, membrane and cell wall biosynthesis, enabled independent escape of the first archaebacterial and eubacterial cells from their hydrothermal hatchery, within which the LUCA itself remained [81, 82]. A concept of LUCA growing on the H_2_/CO_2_ couple, and being naturally chemiosmotic is among many other hypotheses. This point goes a long way towards explaining why chemiosmosis, and the proteins that harness ion gradients, are universal among living cells [83]. LUCA could have used proton gradients to drive carbon and energy metabolism, but only if the membranes were leaky. This requirement precluded ion pumping and the early evolution of phospholipid membranes [84]. However, other researchers demonstrated in an evidence-based manner that LUCA was enclosed by a lipid membrane with secretory and insertion apparatus of protein nature. Comparative genomic analyses showed that LUCA already encoded several critical membrane-bound proteins [85, 86] as well as ATP-ase, contained ribosomes and most likely DNA [28, 87]. These sophisticated ribosomes of LUCA were built of 34 proteins that are shared by all ribosome-encoding organisms [8, 88]. The following issue after deciphering LUCA’s nature in tracing early cellular evolution is to explain the differences in the membrane composition (cytoplasmic, nuclear and belonging to reticulum) among the three major domains of life that came after LUCA. Eukarya and Bacteria are much more similar to each other in this regard than Archaea. Eukaryan lipids are bacteria-like and have an opposite chirality as compared to Archaea [85]. Two viruses with related DNA replication systems could have infected RNA cells with different types of lipids, and some cellular lineages ended up using specifically one of the two types of lipids to produce Archaea and Eukarya [61].

**Fig. 2:**
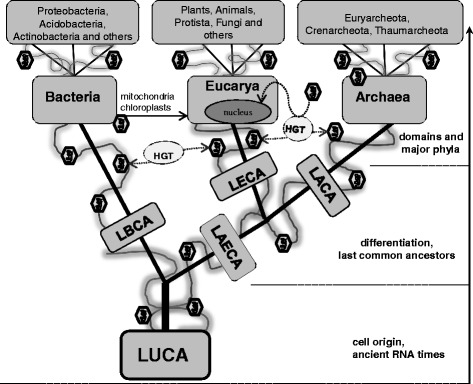
Tree of life. Schematic presentation of the tree of life. Viruses are depicted as small hexagons. Viral lineages are traced as “lianas” wrapping around the trunk and three major branches - domains of life. Horizontal gene transfer (HGT) between cells and viruses is marked as a source of genetic diversity. Viral origin of eukaryal nucleus and bacterial origin of mitochondria and chloroplasts are depicted. Only chosen phyla are presented on the top of the tree. The taxonomy of Archaea is presented according to Brochier-Armanet et al. [91]. For a more detailed taxonomy of major phyla in Eukarya and Bacteria one may refer to Zhao et al. [92], and Chun et al. [93], respectively

Viruses can be considered as living organisms only when they redirect cellular metabolism to reproduce virions, hence infection transforms the ribocell (cell encoding ribosomes and dividing by binary fission) into a virocell (cell producing virions) or ribovirocell (cell that produces virions but can still divide by binary fission) [4, 5]. This nomenclature is in line with a well documented observation of a variety of nonrelated viruses inducing a recruitment of organelles, usually to the perinuclear area, and building a new structure called “virus factory” that functions in viral replication, assembly, or both. The virus factory is enclosed by a membrane, contains ribosomes and cytoskeletal elements and it can also recruit mitochondria, from which it obtains ATP [89]. At this stage of NCLDVs replication cycle the virus factory is very similar to small unicellular parasites such as bacteria. From this perspective it is much easier to consider NCLDVs as entities linking inert world and living cells. Another interesting aspect is that a large poxvirus-like dsDNA virus might be at the origin of the eukaryotic nucleus, enclosed by an ancestral cell and adapted as an organelle. This virus factory in ancient times was very similar to a “viral nucleus” that could have evolved into a modern eukaryotic nucleus according to eukaryogenesis hypothesis [90]. The nucleus could have already appeared in a RNA LUCA and two independent transfers of DNA from viruses to cells were suggested to explain the existence of two nonhomologous DNA replication machineries – one in Bacteria, the other in Archaea and Eukarya, which for that reason are placed on a common branch of the tree of life as opposed to Bacteria (Fig. 2) [91–93].

Later, it was proposed that DNA replication machineries of each domain could have also originated from three different viruses that helped create three major branches of life: LACA – last archaeal common ancestor, LBCA – last bacterial common ancestor, and finally LECA – last eukaryotic common ancestor [61]. This concept of polyphyletic ancestry of viruses is called “Three RNA cells, three DNA viruses”. It is interesting to denote that RNA viruses might have been at the origin of DNA biochemistry. RNA-based viruses replicating in RNA-based cells would have acquired an RNA-to-DNA modification system to resist cellular RNA-degrading enzymes (Darwinian selection). For this to happen, RNA viruses acquired the ribonucleotide reductase for conversion of diphosphate-ribonucelotides to diphosphate-deoxyribonucelotides, and thymidylate synthase to make dTMP from dUMP, cellular RNA was then replaced by DNA of possible viral origin in the course of evolution [57]. The genetic DNA-RNA takeover may have been driven by a combination of increased chemical stability, increased genome size and irreversibility as it was demonstrated experimentally several years ago [94]. This scenario is supported by the fact that many modern viruses encode viral-specific versions of ribonucleotide reductases and thymidylate synthases. Interestingly, to further support the above, deoxyuridine is known to replace thymidine in the DNA of several bacteriophages [95]. Given the complexity of ribosomes and sophisticated nature of aforementioned enzymes it would be really difficult to imagine that they originated in the world without proto-cells. The RNA-to-DNA transition must have taken place in a cellular context [60].

## Conclusion and future perspectives

It is legitimate to say that the tree of life is composed of cells/organisms coding for ribosomes and multiplying by binary fission, and viruses are excluded from the tree of life (cells) as entities encoding capsid proteins and undergoing intracellular process in order to propagate [9]. They are actually molecular genetic parasites. All life must survive this viral-laden habitat and survivors generally retain prophage (or provirus) or their defectives [96]. Viruses from the very origin of life were one of major sources of global genetic biodiversity by participation in altering genomic structures (mutations) and functions. They also served as vehicles to transfer host genes horizontally between cells from different species and even distant taxa. From the time of LUCA, viruses have coevolved with their hosts, and citation by Forterre seems appropriate: “viral lineages can be viewed as lianas wrapping around the trunk, branches and leaves of the tree of life” [4]. Koonin ventures to postulate that the concept of the tree of life should be replaced by a “complex network of treelike and netlike routes of evolution to depict the history of life”, which indeed may better reflect reality [97]. For decades virologists have tried to understand and explain the origin of viruses. In our opinion, we probably need to cope with the idea that all concepts on virus origin described in this review are complementary. Viroids and HDV by their nature may support the co-evolution theory by the former and the escape concept by the latter. A recent discovery of mimivirus (more or less a decade ago) and other giant viruses has opened for some scientists a new perspective on evolution of these viruses from an extinct fourth cellular domain. However, the concept of this new domain is very controversial in the scientific community [98]. This late discovery of giruses is a good example of how difficult it was to leave a dogma of viruses being the smallest entities passing through the finest filters as opposed to bacteria [99, 100] and that perhaps some new great discoveries of the viral world are still ahead of us. Viruses are especially neglected in phylogenetic studies because they lack a unifying genetic marker, similar to rRNA for cells and because their genetic activity is underestimated [101]. The ancestors of current life forms no longer exist and it makes extremely difficult to go back to the dawn of evolution that took place several billion years ago. Paradoxically, future findings in the viral world and even more powerful tools of bioinformatics for comparative studies may help better understand the first evolutionary events.

## Abbreviations

SMA: Supramolecular aggregates
REO: Ribosome-encoding organisms
CEO: Capsid-encoding organisms
LACA: Last universal common ancestor
LUCAS: Last universal common ancestor state
LAECA: Last archaeal-eukaryal common ancestor
LACA: Last archaeal common ancestor
LBCA: Last bacterial common ancestor
LECA: Last eukaryotic common ancestor
HGT: Horizontal gene transfer
LGT: Lateral gene transfer
ORF: Open-reading frame
NCLDV: Nucleo-cytoplasmic large DNA viruses
ORFan: Orphan genes
HDV: Hepatitis delta virus
HBV: Hepatitis B virus
APMV: *Acanthamoeba polyphaga* mimivirus
